# Disseminated cryptococcosis in a renal transplant recipient

**DOI:** 10.1002/rcr2.1011

**Published:** 2022-07-31

**Authors:** Seira Ozawa, Shigeo Hanada, Hironori Uruga, Hiroyo Ito, Hiroshi Nakahama, Narishige Ishikawa, Shuhei Moriguchi, Kyoko Murase, Atsushi Miyamoto, Nobukazu Hayashi, Daiya Takai

**Affiliations:** ^1^ Department of Respiratory Medicine, Respiratory Center Toranomon Hospital Tokyo Japan; ^2^ Okinaka Memorial Institute for Medical Research Tokyo Japan; ^3^ Department of Pathology Toranomon Hospital Tokyo Japan; ^4^ Department of Dermatology Toranomon Hospital Tokyo Japan

**Keywords:** disseminated cryptococcosis, lung nodules, papule, renal transplant recipient

## Abstract

Skin cryptococcosis often manifests as an umbilicated papule, and chest computed tomography findings of multiple nodules and cavities are also characteristic. The combination of characteristic cutaneous manifestations and radiological findings can help clinicians make an “at‐a‐glance” diagnosis of disseminated cryptococcosis.

## Graphical abstract

A papulonodular lesion with an umbilicatedcenter presented on the nasalbridge and multiple nodules with cavitary lesions on chest computed tomographyare very impressive. Grocott stain of skin biopsy specimen shows numerousyeastlike organisms, suggesting a cutaneous manifestation of disseminated cryptococcosis.

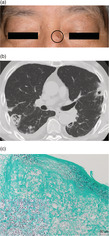

## CLINICAL IMAGE

**FIGURE 1:**
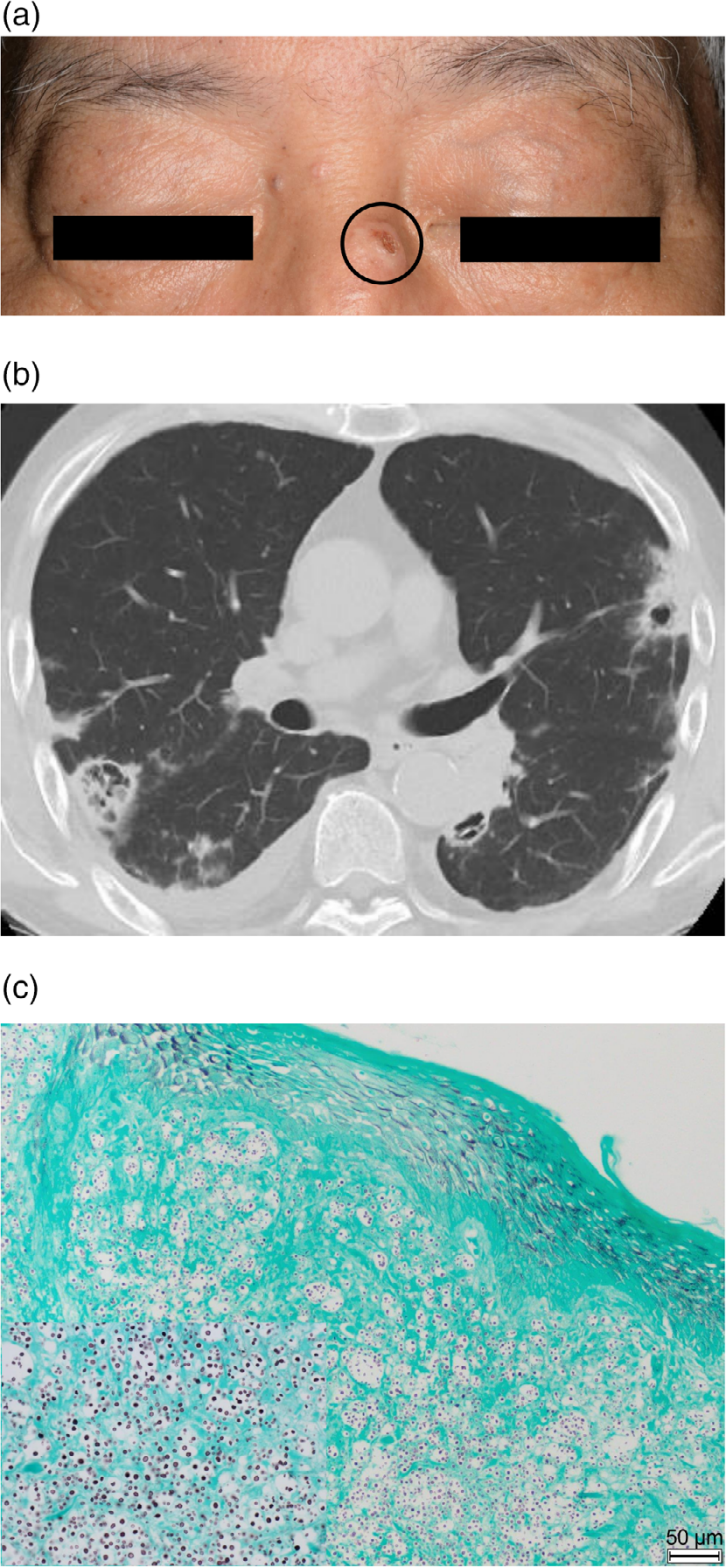
A papulonodular lesion with an umbilicated center, resembling molluscum contagiosum, is present on the nasal bridge (A). Chest computed tomography shows multiple nodules, cavitary lesions, infiltrations with air bronchograms, and a pleural effusion (B). Grocott staining of a skin biopsy specimen shows numerous yeastlike organisms surrounded by mucinous capsules and budding on histology (C). The 50 μm bar shows the scale.

A 64‐year‐old man presented with a 2‐week history of chest pain and dyspnea on exertion. He had undergone renal transplantation due to immunoglobulin A nephropathy 14 months previously. His medications included methylprednisolone, tacrolimus and mycophenolate mofetil. An umbilicated brown papule was noted on his nasal bridge (Figure 1A). Chest computed tomography revealed multiple nodules, infiltrations with air bronchograms, cavitary lesions and a pleural effusion (Figure 1B). Laboratory testing revealed a serum cryptococcal antigen dilution titre of 1:16384 (reference: <1:2). Further, *Cryptococcus neoformans* was grown on sputum culture. A skin biopsy of the nasal lesion showed numerous yeast forms with thick capsules and budding on histology (Figure 1C), confirming the diagnosis of disseminated cryptococcosis. The cutaneous and pulmonary lesions improved following treatment with liposomal amphotericin B and flucytosine. Cryptococcosis is the third most common invasive fungal infection in solid organ transplant recipients,^1^ and is a significant cause of morbidity and mortality. Skin cryptococcosis often manifests as an umbilicated papule.^2^ The combination of characteristic cutaneous manifestations and radiological findings can help clinicians make an “at‐a‐glance” diagnosis. If clinicians suspect secondary cryptococcosis in a skin lesion as a complication of disseminated disease, diagnostic evaluation and optimal targeted therapy should be provided immediately.

## AUTHOR CONTRIBUTION

All authors provided patient care. Seira Ozawa, Shigeo Hanada and Daiya Takai drafted and edited manuscript and selected the images. All authors contributed to interpretation of the data, and critically reviewed and approved the final version of the manuscript.

## CONFLICT OF INTEREST

None declared.

## ETHICS STATEMENT

The authors declare that appropriate written informed consent was obtained for the publication of this manuscript and accompanying images, including the photograph of the patient's face.

## Data Availability

Data sharing not applicable to this article as no datasets were generated or analysed during the current study.
